# Association between duration of antipseudomonal beta-lactam therapy and *Clostridioides difficile* infections in monomicrobial *Enterobacterales* bloodstream infections at an academic medical center

**DOI:** 10.1017/ash.2022.3

**Published:** 2022-02-18

**Authors:** Audry M. Hawkins, Brian Raux, Erin Weeda, Krutika Mediwala Hornback

**Affiliations:** 1 Franciscan Health Mooresville, Mooresville, Indiana; 2 Department of Pharmacy, Medical University of South Carolina (MUSC) Health, Charleston, South Carolina; 3 Department of Clinical Pharmacy & Outcome Sciences, Medical University of South Carolina, Charleston, South Carolina

## Abstract

**Objective:**

To evaluate the effects early de-escalation of antipseudomonal β-lactam (APBL) on 90-day CDI risk in *Enterobacterales* bloodstream infections (BSIs).

**Design:**

Retrospective cohort analysis.

**Setting:**

An academic medical center in South Carolina.

**Patients:**

We included patients aged >18 years with monomicrobial BSIs with *Enterobacterales* who received APBL between July 1, 2015, and June 30, 2020.

**Methods:**

Rates of CDI were compared between patients who received an APBL for >72 hours and <72 hours, followed by comparison between formulary APBLs utilized.

**Results:**

In total, 447 patients were included; 292 and 155 patients received APBL for < 72 hours and > 72 hours, respectively. The incidences of CDI for <72 hours compared to >72 hours were 2.4% and 6.5%, respectively (unadjusted hazard ratio [HR], 2.70; 95% confidence interval [CI], 1.03–7.10; *P* = .04). This difference was not statistically significant in the adjusted model (HR, 2.66; 95% CI, 0.97–7.31; *P* = .06). Meropenem was associated with an increased risk of CDI when compared with all other formulary APBLs: 4 (26.7%) of 15 versus 13 (3.0%) of 432 (*P* < .001).

**Conclusions:**

Utilization of an APBL for >72 hours was associated with a statistically significant increase in the incidence of CDI in an unadjusted model and with a numerically higher CDI incidence in the adjusted model. Meropenem was the formulary APBL that carried the highest risk of CDI. The results of this study provide further evidence supporting active antimicrobial stewardship to reduce unnecessary broad-spectrum antibiotics in the effort to alleviate the burden that CDI imposes on the healthcare system.

*Clostridioides difficile* infections (CDI) are considered a “major health threat” in the United States, according to the Centers for Disease Control and Prevention (CDC).^1^ CDI can occur as a result of misuse of broad-spectrum antibiotics, providing further evidence in support of active antimicrobial stewardship (AMS).

Antipseudomonal β-lactams (APBLs) have gained increasing attention as a major contributor to CDI.^2^ In a recent study, empiric use of APBL for >48 hours was an independent risk factor for CDI.^3^ Despite the rarity of *P. aeruginosa* BSI in the absence of immunodeficiency, excessive use of APBLs continues.^4,5^ De-escalation is critical in preventing bacterial resistance, as well as healthcare-associated CDI.^6,7^

Currently, the Medical University of South Carolina (MUSC) Health utilizes rapid diagnostic testing (RDT) to identify organisms in positive blood cultures in as little as 24 hours. Although this technology is utilized in the laboratory, the stewardship implications of the results may be difficult to interpret. However, they have shown mortality benefit when combined with active antimicrobial stewardship programs (ASPs).^8,9^ The goal of this study was to gather data that support active antimicrobial stewardship and utilization of RDT to de-escalate APBL therapy for *Enterobacterales* BSIs. In this retrospective cohort analysis, we aimed to elucidate the effects of prolonged APBL treatment by comparing CDI rates associated with durations of either >72 hours or ≤72 hours.

## Methods

### Setting

This analysis was conducted at the Charleston campus of Medical University of South Carolina Health. This campus contains 2 acute-care adult hospitals, with ∼800 beds.

### Definitions

Monomicrobial BSI was defined as having 1 species from the *Enterobacterales* family in the index blood culture. The primary source was defined according to the CDC criteria.^10^ The initial APBL was the APBL that was started on the date of the index blood culture. Concomitant antimicrobials were those other than an APBL utilized during treatment. Duration of therapy was defined in hours. The primary definitive agent was the antibiotic utilized for the longest period prior to de-escalation, discontinuation, or completion of therapy. Formulary APBLs included piperacillin-tazobactam, cefepime, ceftazidime, meropenem, imipenem-cilastatin, and aztreonam. CDI was defined as a laboratory diagnosis from *C. difficile* toxin polymerase chain reaction (PCR) testing via standalone PCR (Cepheid, Sunnyvale, CA) or the gastrointestinal (GI) panel PCR (BioFire Diagnostics, Salt Lake City, UT) prior to October 2019. After October 2019, the standalone PCR was removed and a 2-step algorithm of PCR with reflex of positives to toxin enzyme immunoassay (EIA) test (C diff Quik Chek Complete, TechLab, Blacksburg, VA) became the primary mode of CDI diagnosis. The GI PCR can still be ordered. Concomitant CDI and BSI was defined as having a positive *C. difficile* PCR within 24 hours of index blood culture collection.

### Microbiology techniques

Identification of bloodstream organism isolates is routinely done via the BioFire FilmArray Blood Culture Identification system (BCID, BioFire Diagnostics, Salt Lake City, UT). When an organism was not detected via BCID, matrix-assisted laser desorption-ionization-time of flight (MALDI-TOF, Bruker, Billerica, MA) was used to test organism growth. CDI was defined as a laboratory-based diagnosis made via standalone PCR, GI PCR, or a 2-step algorithm of PCR with reflex of positives to toxin EIA test.

### Case selection

Data were acquired for patients aged ≥18 years who had a monomicrobial BSI identified by a positive *Enterobacterales* blood culture result and received APBL between July 1, 2015, and June 30, 2020. Patients were excluded if any of the following were true: CDI preceding BSI and treatment with APBL, CDI >90 days after BSI, CDI within past year, concomitant CDI and BSI, polymicrobial BSI, or hospital discharge prior to 72 hours of therapy. Included patients were stratified into 2 groups based on the total hours of APBL therapy received, APBL ≥72 hours or <72 hours. CDI was measured during a 90-day period starting the first date APBL was administered. Sensitivity analyses that excluded those diagnosed by a method other than standalone PCR were conducted because standalone PCR was the method of diagnosis most utilized by our institution during the study period. No other microbiological findings were assessed in this study; thus, appropriateness of antibiotics was beyond the scope of this study.

### Statistical analysis

Categorical variables are reported as frequencies with percentages and were compared between cohorts using the χ^2^ test or the Fisher exact test. Continuous variables were reported as medians with interquartile ranges and were compared between cohorts using the Wilcoxon rank-sum test. To assess the primary objective of CDI in patients who received either >72 hours or <72 hours of APBL, hazard ratios (HR) and 95% confidence intervals (CI) were estimated using Cox proportional hazards regression. An adjusted HR was calculated after accounting for clinical considerations (ie, Pitt bacteremia score, length of stay prior to the BSI, and Charlson comorbidity score) carrying the potential to increase the patient’s risk for CDI according to previous literature.^3^ Statistical analyses were conducted using SPSS version 25 software (IBM, Armonk, NY), and *P* values <.05 were considered significant. The Institutional Review Board of MUSC Health deemed this a quality improvement project and waived the need for oversight.

## Results

Among 502 patients identified, 55 patients were excluded. Of those 55 patients, 28 patients with CDI preceding BSI and treatment with APBL were excluded; 7 patients with CDI >90 days after BSI were also excluded. Furthermore, 6 patients had CDI within the past year and 6 had concomitant BSI, leading to exclusion. Patients with polymicrobial BSI (n = 7) or hospital discharge prior to 72 hours of therapy (n = 1) were excluded. Of the 447 patients remaining, 292 patients received APBL for ≤72 hours and 155 patients received APBL for >72 hours (Table 1). The 17 patients who developed CDI were compared with 430 patients who did not. Overall, the median age was 62 years, and most 255 patients (57%) were male. An intra-abdominal infection source occurred most frequently (n = 173, 38.7%). We detected no statistically significant differences in baseline characteristics.

**Table 1.** Characteristics of Included Patients with *Enterobacterales* Bloodstream Infections

Variable	APBL≤72 Hours (N=292) No. (%)^a^	APBL>72 Hours (N=155) No. (%)^a^	*P* Value
Age, median y (IQR)	62 (51–71)	62 (51–72)	.87
Sex, female	129 (44)	63 (41)	.47
**Race**			
White	155 (53)	72 (48)	.27
Black	124 (43)	78 (50)	
Other	13 (5)	5 (3)	
Any documented allergy	177 (61)	90 (58)	.60
ALT, median (IQR)	33 (20–75)	34 (17–60)	.28
AST, median (IQR)	41 (26–87)	40 (23–73)	.20
Chronic kidney disease	68 (23)	43 (28)	.30
Diabetes	91 (31)	55 (36)	.35
Indwelling catheter	95 (33)	54 (35)	.62
Skilled nursing facility resident	12 (4.1)	8 (5.2)	.61
Intraabdominal source of infection	118 (40)	55 (36)	.31
Empirical combination therapy	98 (34)	53 (34)	.89
Prior antibiotics in last 3 months	121 (41)	56 (36)	.28
Primary definitive therapy with ceftriaxone or fluoroquinolones	49 (17)	22 (14)	.48
Charlson comorbidity index, median (IQR)	5 (3–8)	6 (3–8)	.33
LOS prior to BSI ≥2 days	77 (26)	48 (31)	.30
**Pitt bacteremia score**			.26
0	157 (54)	71 (46)	
1–2	69 (24)	41 (27)	
≥3	66 (23)	43 (28)	

Note. APBL, antipseudomonal β-lactam; BSI, bloodstream infection; IQR, interquartile range; LOS, length of stay.

a Units unless otherwise specified.

Within 90 days of BSI and receipt of APBL, 17 patients developed CDI. Of the patients diagnosed with CDI in our study, diagnosis occurred via standalone PCR in 14 patients (82.4%), via GI PCR in 2 patients (11.8%), and via PCR plus EIA in 1 patient (5.9%). The median time to CDI was 9 days (interquartile range [IQR], 5–25 days). When stratifying time to CDI by method of CDI diagnosis, time to standalone PCR was 22 days (IQR, 7–47 days), time to GI PCR was 12 days for one patient and 77 days for the other, and PCR + EIA was 7 days. CDI occurrence in patients receiving APBL for <72 hours was 2.4% compared to 6.5% in patients receiving APBL for ≥72 hours (hazard ratio [HR], 2.70; 95% CI, 1.03–7.10; *P* = .04). After adjusting for the clinical characteristics previously mentioned, CDI incidence was no longer statistically different between groups (HR, 2.66; 95% CI, 0.97–7.31; *P* = .06) (Table 2). Results were similar (HR, 2.51; 95% CI, 0.82–7.70; *P* = .11) upon sensitivity analysis (ie, when those 3 cases that were diagnosed by a method other than standalone PCR were excluded).

**Table 2.** Multivariable Cox Regression Analysis Evaluating the Association between Antipseudomonal β-Lactam (APBL) Duration and *Clostridioides difficile* Infection

Model	Hazard Ratio (95% CI)	*P* Value
Unadjusted Model, APBL >72 h	2.70 (1.03–7.10)	.04
Adjusted Model, APBL>72 h^a^	2.66 (0.97–7.31)	.06

Note. CI, confidence interval.

a Analysis was adjusted for Pitt bacteremia score, length of stay prior to the bloodstream infection, and Charlson comorbidity score.

The APBL agents utilized were cefepime, meropenem, and piperacillin-tazobactam (Table 3). Among them, meropenem was associated with higher rates of CDI when compared with all other formulary APBL: 4 (26.7%) of 15 versus 13 (3.0%) of 432 (*P* < .001). After excluding the 3 patients diagnosed by a method other than the standalone PCR, the association between meropenem and a higher occurrence of CDI remained: 4 (26.7%) of 15 versus 10 (2.3%) of 429 (*P* < .001).

**Table 3.** Empiric Antipseudomonal β-Lactam Utilization

Agent	Total (N=447), No. (%)
Cefepime	65 (14.5)
Meropenem	15 (3.6)
Piperacillin-tazobactam	367 (82.1)

## Discussion

This analysis demonstrates that the receipt of APBL for >72 hours is a potential risk factor for CDI. Previous studies evaluating cumulative antibiotic exposure effects on CDI assessed durations ranging from minimal perioperative antibiotic exposures to antibiotic exposures >7 days.^11–13^ Thabit et al^14^ found varying median times to onset of CDI among their patient population, with meropenem having the fastest median time of onset to CDI, occurring ∼6 days after initial receipt. Cefepime utilization increased risk of CDI regardless of discontinuation. Receiving piperacillin-tazobactam was associated with the longest median time of onset to CDI in this study, occurring past 14 days.^14^ In our patient population, 72 hours was chosen to demonstrate the effects of early de-escalation while accounting for institutional microbiology laboratory practices, transcription of results into the electronic medical record, provider interpretation, and ASP intervention.

Meropenem appeared to increase the risk of CDI in our patient population. The increased risk of CDI with carbapenems, relative to other APBLs, has been previously established.^15^ Despite its in vitro activity against *C. difficile* strains, meropenem has not been proven protective against CDI.^15,16^ Similar to Lee et al,^15^ patients in our study receiving piperacillin-tazobactam had numerically lower rates of CDI compared to both cefepime and meropenem: 2.45% vs 4.62% vs 26.67%, respectively. Researchers in the aforementioned studies hypothesized that patients receiving carbapenems had concomitant risk factors for CDI; however, evaluation of baseline characteristics was outside the scope of this study.

Lew et al^17^ evaluated how ASP-guided carbapenem de-escalation affects clinical success and adverse effects. Once de-escalation to a noncarbapenem occurred, a statistically significant decrease in antibiotic-associated diarrhea and numerically lower rates of CDI was demonstrated. Median time to de-escalation was 6 days in this study, and >50% of interventions occurred after antimicrobial susceptibility testing was completed.^17^ As previously noted, MUSC Health utilizes RDT for blood culture identification. AMS practices at our institution heavily integrate RDT microbiology techniques into ASP via infectious disease pharmacist–driven blood-culture review and participation in microbiology technical rounds. This procedure streamlines stewardship actions and increases dissemination of information on the utility of these tests. RDT paired with ASP is a well-supported, data driven method for antimicrobial de-escalation. MacVane et al,^20^ in a study previously conducted at our institution, demonstrated that the addition of RDT to ASP led to shorter times to appropriate therapy and higher rates of antimicrobial de-escalation.^18–20^ These results, paired with our findings, emphasize the importance of active AMS initiatives focused on RDT interpretation to aid in early de-escalation in the effort to decrease overall rates of CDI.

This study had several limitations. First, given the low number of CDI events, our study may have been underpowered despite rates of CDI similar to those of like patient populations previously described.^3^ Second, changes in institutional diagnosis of CDI occurred during the study period. Limitations of PCR only diagnostics for CDI have been previously defined, potentially leading to overdiagnosis of CDI.^21^ Appropriateness of CDI testing and treatment, as well as antibiotic appropriateness, were beyond the scope of this study.

In summary, APBL utilization for >72 hours was associated with an increased risk of CDI in our unadjusted model. The adjusted model demonstrated a numerical increase in CDI among patients receiving >72 hours of APBL and is consistent with prior findings. The effect of meropenem on CDI in our patient population further underscores the importance of careful empiric antimicrobial selection and active ASP paired with RDT guided de-escalation in patients with *Enterobacterales* BSIs.
